# Facilities for Medical Instruction at London, England

**Published:** 1850-08

**Authors:** 


*Facilities for Medical Instruction at London, England.*—Dr. Smith, Editor of the Boston Medical and Surgical Journal, who is travelling in Europe for professional observation, and the restoration of health, is furnishing for his readers some highly interesting letters respecting medical matters abroad. His tour, thus far, has extended only through Great Britain. In a late letter he speaks of the comparative advantages for medical instruction at London, and in the United States, as follows:

"After a careful examination, it may be said that with all the presumed and admitted advantages for medical study in this magnificent capital, the facilities in most respects are quite equal in Boston, Philadelphia and New York. A more preposterous apology for going abroad, so far as England is concerned, was never devised. It is susceptible of demonstration that American students are more industrious at home than on their arrival in Europe. At the moment of writing, it has been stated, on reliable authority, that anatomical pursuits are conducted here with extreme difficulty, on account of the scarcity and expense of subjects. When the limbs retail for ten shillings apiece, poor students become economical of *materiel.* Not an individual dying in a hospital is taken for the theatre. Prisons and work-houses are exclusively the sources of supply. If friends claim the body, the schools cannot be supplied with it."

